# Supplementary material for: Complexome profiling of the Chlamydomonas psb28 mutant reveals TEF5 as an early PSII assembly factor
Source: Plant Cell. 2025 Mar 17;37(6):koaf055. doi: 10.1093/plcell/koaf055 (PMC12164590; doi:10.1093/plcell/koaf055)
Supplement: koaf055_Supplementary_Data [file koaf055_supplementary_data.zip › psb28_interactive.zip!/plots/intensity/qualityPlots/peptides/ATPVB_Cre02.g076350.t1.2.html]

### Cre02.g076350.t1.2

Trivial name: ATPVB  
SeqCoverage [median]: 38.30  
Mass: 55.80 kDa  
  
 

| Sequence | MissedCleavage | Mass [Da] | wtIntensity | muIntensity | Score |
| --- | --- | --- | --- | --- | --- |
| AVDALSSELAFK | 0 | 1249.66 | 12289.07 | 26346.41 | 173.37 |
| AVVQVFEGTSGIDNR | 0 | 1590.80 | 5469.45 | 20175.79 | 210.93 |
| AVVQVFEGTSGIDNRK | 1 | 1718.90 | 5738.38 | 36967.94 | 143.38 |
| EEVPGRR | 1 | 841.44 | 0.00 | 601.62 | 82.26 |
| FVAQGPYENR | 0 | 1179.57 | 2459.45 | 17454.52 | 141.48 |
| GQVLEVDGTR | 0 | 1072.55 | 2391.49 | 14384.16 | 105.20 |
| GYPGYMYTDLATIYER | 0 | 1911.87 | 1293.10 | 1047.69 | 96.10 |
| KDHSEVSNQLYANYAIGK | 1 | 2036.00 | 0.00 | 1915.71 | 60.31 |
| KFVAQGPYENR | 1 | 1307.66 | 393.34 | 4389.67 | 123.01 |
| KTSLEFTGEVLTTPVSR | 1 | 1863.99 | 1834.51 | 13530.97 | 64.51 |
| LGDGSLR | 0 | 716.38 | 0.00 | 4230.45 | 84.70 |
| QDFEENGSLER | 0 | 1322.57 | 0.00 | 697.09 | 68.85 |
| QIYPPINVLPSLSR | 0 | 1595.90 | 2590.00 | 0.00 | 92.87 |
| RGQVLEVDGTR | 1 | 1228.65 | 4967.41 | 15628.46 | 84.29 |
| SAIGEGMTR | 0 | 920.44 | 0.00 | 4928.60 | 82.45 |
| TSLEFTGEVLTTPVSR | 0 | 1735.90 | 1508.91 | 658.39 | 132.32 |
| TVLFLNLANDPTIER | 0 | 1714.93 | 7555.73 | 7761.17 | 185.25 |
| TYPEEMIQTGISTIDVMNSIAR | 0 | 2468.19 | 2229.39 | 16167.34 | 81.58 |
| YAEIVEIR | 0 | 991.53 | 14466.16 | 54670.42 | 138.90 |
|  |  |  | 65186.4 | 241556.4 |  |
